# Aqueous Extract of Semen* Ziziphi Spinosae* Exerts Anxiolytic Effects during Nicotine Withdrawal via Improvement of Amygdaloid CRF/CRF1R Signaling

**DOI:** 10.1155/2018/2419183

**Published:** 2018-09-02

**Authors:** Changhong Gu, ZhengLin Zhao, Xiaodong Zhu, Tong Wu, Bong Hyo Lee, Yu Jiao, Chul Won Lee, Dae Hwa Jung, Chae Ha Yang, Rongjie Zhao, Sang Chan Kim

**Affiliations:** ^1^Research Institute of Medicine and Pharmacy & College of Mental Health, Qiqihar Medical University, Qiqihar 161006, China; ^2^Medical Research Center, College of Oriental Medicine, Daegu Haany University, Gyeongsan-si 38610, Republic of Korea; ^3^Department of Pharmacology, Mudanjiang Medical University, Mudanjiang 157011, China

## Abstract

Anxiety during nicotine withdrawal (NicW) is a key risk factor for smoking relapse. Semen* Ziziphi Spinosae* (SZS), which is a prototypical hypnotic-sedative herb in Oriental medicine, has been clinically used to treat insomnia and general anxiety disorders for thousands of years. Thus, the present study evaluated the effects of the aqueous extract of SZS (AESZS) on NicW-induced anxiety in male rats that received subcutaneous administrations of nicotine (Nic) (0.4 mg/kg, twice a day) for 7 d followed by 4 d of withdrawal. During NicW, the rats received four intragastric treatments of AESZS (60 mg/kg/d or 180 mg/kg/d). AESZS dose-dependently attenuated NicW-induced anxiety-like behaviors in the elevated plus maze (EPM) tests and 180 mg/kg/d AESZS inhibited NicW-induced increases in plasma corticosterone. Additionally, the protein and mRNA expressions of corticotropin-releasing factor (CRF) and CRF type 1 receptor (CRF1R) increased in the central nucleus of the amygdala (CeA) during NicW, but these changes were suppressed by 180 mg/kg/d AESZS. A post-AESZS infusion of CRF into the CeA abolished the attenuation of anxiety by AESZS and 180 mg/kg/d AESZS suppressed NicW-induced increases in norepinephrine and 3-methoxy-4-hydroxy-phenylglycol levels in the CeA. The present results suggest that AESZS ameliorated NicW-induced anxiety via improvements in CRF/CRF1R and noradrenergic signaling in the CeA.

## 1. Introduction

Tobacco smoking is a leading preventable cause of disease and death worldwide [1]. Nicotine (Nic) is the primary psychoactive and addictive component of tobacco and dependence on this drug is the main cause associated with continued smoking [2]. It has been estimated that most smokers attempt to quit using tobacco but few of them succeed due to the discomforts associated with Nic withdrawal (NicW) symptoms [3]. Of the various NicW symptoms, increased anxiety appears to be the most prominent because it is the most commonly reported NicW symptom among smokers [4] and has been well documented in a variety of animal tests [5]. Therefore, there is a growing interest in the development of novel and safe medications that can prevent or relieve the anxiety experienced during NicW so that smokers persist in the attempt to quit.

Withdrawal-induced anxiety during the cessation of drugs of abuse, including Nic, is derived from dysregulation of brain stress systems which is characterized by escalated activities of corticotropin-releasing factor (CRF) and norepinephrine (NE) systems in brain regions involved in emotional processing [6, 7].

The central nucleus of the amygdala (CeA) is a primary structure associated with emotional memory mediating the generation and expression of anxiety [6]. The CeA is innervated by CRFergic neurons, and dysregulation of the CRF system in the CeA is closely related to the withdrawal-induced anxiety associated with drugs of abuse. For example, withdrawal from cannabinoids and cocaine enhances CRF release in the CeA [8, 9] and withdrawal from ethanol and Nic increases CRF mRNA expression in the CeA [10, 11]. In contrast, antagonizing CeA CRF signaling attenuates withdrawal-induced anxiety and reduces drug-seeking behaviors [12]. NE is a secretagogue of CRF [13] and the CeA receives heavy noradrenergic projections from the brainstem [14]. It is known that withdrawal from drugs of abuse significantly upregulates noradrenergic activities in the CeA [7]. For example, cocaine withdrawal elevates *β*1-adrenergic receptor expression in the CeA [15], while treatment with betaxolol, which is a selective *β*1-adrenergic receptor antagonist, mitigates cocaine withdrawal-induced anxiety [16]. Ethanol withdrawal increases NE synthesis and utilization in the CeA, and the inhibition of tyrosine hydroxylase can block the occurrence of withdrawal-induced anxiety [17, 18]. Hence, the CRF and NE systems in the CeA represent promising neurochemical targets for the development of novel medications that can treat anxiety induced by withdrawal from drugs of abuse.

Semen* Ziziphi Spinosae* (SZS), or the seeds of* Zizyphus jujuba Millervar. spinosa Hu ex H. F. Chou* (also known as jujube seeds), represent the prototypical hypnotic-sedative herb used in traditional Chinese medicine. Oriental medicine practitioners have used SZS decoctions to clinically treat insomnia and anxiety for thousands of years [19]. Modern scientific analyses have revealed that SZS contains more than 50 bioactive compounds, including saponins, cyclopeptide alkaloids, and C-glycoside flavones, and that some of these compounds exhibit substantial tranquilizing and neuroprotective effects in animals [20]. Spinosin, which is one of the two main SZS compounds stipulated in the Chinese Pharmacopoeia (ChP), potentiates pentobarbital-induced sleep and exerts anxiolytic effects via the GABA and serotonin (5-HT) systems in mice [21–23]. Jujuboside A, which is the other main SZS compound stipulated in ChP, inhibits the hippocampal activities of acetylcholinesterase and ameliorates A*β*_1–42_-induced learning and memory impairments in mice [24]. Magnoflorine is an important cyclopeptide alkaloid inherent in SZS and is known to activate adrenal *β*_2_-receptors and produce anxiolytic and hypnotic effects via central GABAa receptors [25].

Fundamentally, anxiety is the result of inner turmoil within physiological neuroendocrine systems, which is caused by physical, psychiatric, and/or biochemical stressors. Although clinical and preclinical studies have demonstrated the anxiolytic effects of SZS, few studies have explored the effects of SZS on anxiety that is specifically induced by a certain stressor. For example, it has yet to be experimentally determined whether SZS exerts therapeutic effects on withdrawal anxiety induced by the cessation of drugs of abuse. Furthermore, as noted above, studies investigating the neuropharmacological mechanisms underlying SZS have focused on the involvement of GABAergic and serotonergic signaling pathways but have ignored the neuropeptidergic systems in the brain that play pivotal roles in the mediation of anxiety. Therefore, to determine the efficacy of SZS treatment for reducing anxiety associated with Nic dependence, the present study examined the possible anxiolytic effects of SZS during NicW and investigated the relevant mechanisms underlying this process with a focus on the CRF system in the CeA.

## 2. Materials and Methods

### 2.1. Preparation of the Aqueous Extract of SZS

**Figure 1:**
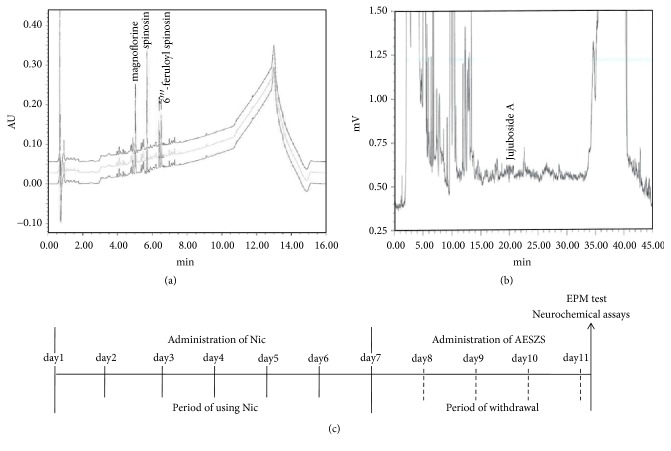
(a) HPLC profile of AESZS, which contains magnoflorine, spinosin, and 6′′′-feruloylspinosin. (b) HPLC profile of AESZS, which contains jujuboside A. (c) Time schedule for NicW, rats were injected with Nic (0.4 mg/kg s.c., twice a day) for 7 consecutive days followed by 4 days of withdrawal and then analyzed for behavioral and neurochemical changes. During NicW, the rats were treated with AESZS (60 or 180mg/kg/d, four times). AESZS: aqueous extract of Semen* Ziziphi Spinosae*; Nic: nicotine; NicW: nicotine withdrawal.

SZS was obtained from Daewon Pharmacy (Daegu, Republic of Korea) and identified by Professor Sang Chan Kim (Daegu Haany University; Daegu, Republic of Korea). The seeds were dry-fried and ground into fine powder. Next, the powdered SZS (200 g) was soaked in distilled water (DW; 2 L) for 30 min at room temperature and extracted at 100°C for an additional 150 min. Then, the solution was filtered through a 0.22-*μ*m syringe filter (Nalgene; NY, USA) and lyophilized in a vacuum evaporator; the yield was 10.62%. Analysis of the aqueous extract of SZS (AESZS) with high-performance liquid chromatography (HPLC) revealed that it contained magnoflorine, spinosin, 6′′′-feruloyl spinosin, and jujuboside A (Figure 1).

### 2.2. Animals and Experimental Design

For this study, 9-week-old male Sprague–Dawley rats weighing 280–300 g were obtained from the Laboratory Animal Center at Qiqihar Medical University (Qiqihar, China). The rats were housed in Plexiglas cages (three rats per cage) in a filtered pathogen-free air environment with a temperature of 21–23°C and a relative humidity of 50%, maintained on a 12:12 h light/dark cycle, and provided with ad libitum food and water. All animal procedures were conducted in compliance with the National Institutes of Health Guide for the Care and Use of Laboratory Animals and were approved by the Animal Care and Use Committee of Qiqihar Medical University.

NicW was induced by administering subcutaneous (s.c.) injections of nicotine hydrogen tartrate (0.4 mg/kg; Sigma; St. Louis, MO, USA) dissolved in saline (pH 7.2; all doses expressed as free base) twice a day for 7 d in their home cages followed by 4 d of withdrawal; the control rats received s.c. administrations of saline. During the NicW period, the rats received intragastric treatments with either DW or AESZS (60 or 180 mg/kg/d, dissolved in DW) once a day for 4 d. At 60 min after the final dose of AESZS (or DW), the rats were subjected to the elevated plus maze (EPM) test to assess anxiety-like behaviors. Immediately following this behavioral test, the rats were euthanized and decapitated, and blood was collected for measurements of plasma corticosterone (CORT) levels. Additionally, the entire brain was removed and stored at -80°C until brain tissue samples were punched from the CeA (anterior–posterior: −2.0 mm, medial–lateral: ± 4.2 mm, and dorsal–ventral: −7.8 mm, based on the Paxinos and Watson rat brain atlas) [26]. The samples were used for Western blot, real-time polymerase chain reaction (PCR), and neurochemical analyses (Figure 1).

### 2.3. EPM Test

At 60 min after the final dose of AESZS, the rats were tested in the EPM to assess anxiety-like behaviors, as previously described [17]. Briefly, the EPM consisted of four arms (50 cm long × 10 cm wide) that were elevated above the ground and monitored with a video tracking system (Ethovision, Noldus Information Technology BV; Wageningen, Netherlands). The two closed arms were enclosed by dark acrylic walls 40 cm in height and the two open arms had ledges 0.5 cm in height. During testing, each rat was placed in the center of the EPM and the numbers of entries into the arms and the time spent in each arm were automatically recorded over a period of 5 min. The percentages of number of entries into the open arms and time spent in the open arms were calculated as follows:

Percentage of Entries_into  open  arms_ = Entries_into  open  arms_/(Entries_into  open  arms_ + Entries_into  closed  arms_) × 100% and

Percentage of Time_spent  in  open  arms_ = Time_spent  in  open  arms_/(Time_spent  in  open  arms_ + Time_spent  in  closed  arms_) × 100%; respectively.

### 2.4. Enzyme-Linked Immunosorbent Assay

For the enzyme-linked immunosorbent assay (ELISA) tests, blood was collected from each rat, mixed with 20 *μ*L of EDTA (20 mg/mL), and then centrifuged at 1500 ×* g* for 10 min at 4°C. Plasma CORT levels were measured using a commercial ELISA kit (Abcam, Cambridge, UK) according to the manufacturers' instructions with the values expressed as ng/mL.

### 2.5. Western Blot Analysis

CeA tissues were homogenized in lysis buffer [20 mM Tris, 5 mM EDTA, 1% Nonidet P-40 (vol/vol), and protease inhibitors] and centrifuged at 16,000 ×* g* for 20 min at 4°C. The proteins in the supernatants were quantified with a bicinchoninic acid (BCA) assay and then subjected to 12% sodium dodecyl sulfate polyacrylamide gel electrophoresis (SDS-PAGE) for separation and transferred onto polyvinylidene difluoride (PVDF) membranes (Millipore; Bedford, MA, USA). The membranes were probed with one of the following primary antibodies: a rabbit polyclonal antibody for CRF (Abcam), a rabbit polyclonal antibody for CRF1R (Abcam), or a rabbit polyclonal antibody for *β*-actin (Abcam). The primary antibodies were tagged with fluorescent IRDye 800CW Goat anti-Rabbit IgG (Li-Cor Bioscience; Lincoln, NE, USA), and the ODYSSEY Infrared Imaging System (Li-Cor Bioscience) was used to read the signals according to the manufacturer's instructions.

### 2.6. Real-Time PCR Analysis

Total RNA was extracted from the CeA tissues with the Trizol reagent (Invitrogen; Carlsbad, CA, USA) and then used to produce cDNA with a reverse transcriptional PCR kit (Promega; Madison WI, USA). Next, a real-time PCR analysis was carried out with a Light Cycler 2.0 (Roche Diagnostics; Mannheim, Germany) using a Light Cycler® DNA Master SYBR green-I kit (Roche) according to the manufacturer's protocol. The primers were synthesized by Bioneer Corporation (Daejeon, Republic of Korea) as follows: 5′-CTCTCTGGATCTCACCTTCCAC-3′ (sense) and 5′-CTAAATGCAGAATCGTTTTGGC-3′ (antisense) for CRF; 5′-GTCTCCAGGGTCGTCTTCAT-3′ (sense) and 5′-CGGACCTCACTGTTCAGAA-3′ (antisense) for CRF1R; and 5′-GTCGTACCACTGGCATTGTG-3′ (sense) and 5′-GCCATCTCTTGCTCGAAGTC-3′ (antisense) for *β*-actin. A housekeeping gene *β*-actin product was used to normalize the given gene expressions and the relative levels were calculated using the following formula:

ΔCT = CT_CRF  or  CRF1R_ − CT_*β*-actin_, ΔΔCT = ΔCT_treated_ − ΔCT_saline_, and presented as 2^−ΔΔCT^.

### 2.7. Intra-CeA Microinfusions

To examine whether the effects of AESZS on NicW-induced anxiety were mediated by amygdaloid CRF, bilateral intra-CeA microinfusions of CRF (0.2 *μ*g/0.2 *μ*L for each side; Sigma Chemical Co.) dissolved in a modified Ringer's solution (MRS; containing 150 mM NaCl, 3.0 mM KCl, 1.4 mM CaCl2, and 0.8 mM MgCl2 in 10 mM phosphate buffer, pH 7.1) were introduced 60 min after the fourth AESZS treatment using a motorized syringe pump (over a period of 60 s). For the intra-CeA infusions of CRF, stainless steel guide cannulae (22-gauge, 2 mm shorter than the 28-gauge injector) were bilaterally implanted into the brain with the cannula tips placed 2 mm above the CeA using a stereotaxic instrument while the rats were anesthetized with sodium pentobarbital [50 mg/kg, intraperitoneal (i.p.)]. Following the surgery, the rats were individually housed in their home cages and given antibiotics (bacitracin ointment and penicillin) and acetaminophen for 3 days to minimize possible infection and pain. After 7 d of recovery from the surgery, the rats underwent the same nicotine withdrawal and drug's treatment schedule. And, at 5 min after the CRF administration, the rats were tested in the EPM.

### 2.8. HPLC Analysis

CeA tissues were weighted and homogenized in 0.1 M perchloric acid (HClO4) and centrifuged at 26,000 × g for 10 min at 4°C. Then, a 20-*μ*L aliquot of supernatant was injected into an HPLC machine with a coulometric detector (Coulochem II, ESA; Bedford, MA, USA) that included a C18 reverse-phase column (5 U ODS, Altex; Ann Arbor, MI, USA) and an electrochemical transducer with a glassy carbon electrode set at 350 mV. The mobile phase was composed of 0.16 M citric acid (pH 3.0), 0.02 mM EDTA with 0.69 mM sodium octanesulfonic acid as an ion-pairing reagent, 4% (v/v) acetonitrile, and 1.7% (v/v) tetrahydrofuran. The peaks and values for NE and 3-methoxy-4-hydroxy-phenylglycol (MHPG) were identified and calculated based on a comparison of their retention times and peak heights with those of standards (Sigma Chemical Co., St Louis, MO, USA); the levels of NE and MHPG were reported as ng/g wet tissue.

### 2.9. Statistical Analysis

All data are expressed as a mean ± standard error of the mean (SEM) and were statistically assessed with one-way analysis of variance (ANOVA) tests followed by Newman-Keuls multiple-comparison tests using the commercially available GraphPad Prism 5.0 software (GraphPad Software; San Diego, CA, USA).* P* values < 0.05 were considered to indicate statistical significance.

## 3. Results

### 3.1. Effects of AESZS on NicW-Induced Anxiety-Like Behaviors

**Figure 2:**
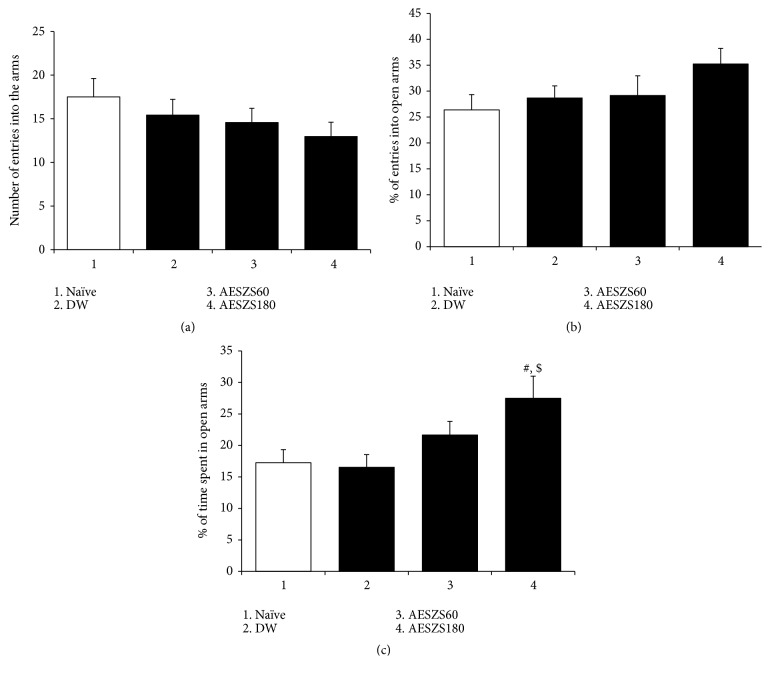
Effects of AESZS on innate anxiety in rats. All data are expressed as a mean ± SEM (*n* = 6). (a) The total number of entries into open and closed arms of the EPM by rats. (b) Percentage of numbers of entries into open arms of EPM by rats. (c) Percentage of time spent in open arms by rats. S: saline, DW: distilled water, AESZS: aqueous extract of Semen* Ziziphi Spinosae*, AESZS60: 60 mg/kg/d AESZS, AESZS180: 180 mg/kg/d AESZS. ^#^*p* < 0.05, versus naive group; ^$^* p *< 0.05, versus DW group (one-way ANOVA followed by Newman-Keuls post hoc test).

A preliminary study was performed to evaluate whether AESZS affected innate anxiety in rats. It revealed that the 180 mg/kg/d dose, but not the 60 mg/kg/d dose, of AESZS administered for 4 d increased the time spent in the open arms of the EPM in naive rats when tested 60 min after the fourth dose [percentage of entries into open arms: F_(3,  20)_ = 1.51,* p *> 0.05,* n* = 6; percentage of time spent in open arms: F_(3,  20)_ = 3.97,* p *< 0.05; naive group versus AESZS180 group,* p *< 0.05; DW group versus AESZS180 group,* p *< 0.05] (Figure 2), which was indicative of improvements in the innate anxiety in the rats. AESZS doses higher than 180 mg/kg/d produced small but evident changes in locomotion, grooming, and nodding in the rats, and therefore, the 60 mg/kg/d and 180 mg/kg/d doses were selected. Another preliminary experiment revealed that saline administration did not change innate anxiety in rats (data not shown).

**Figure 3:**
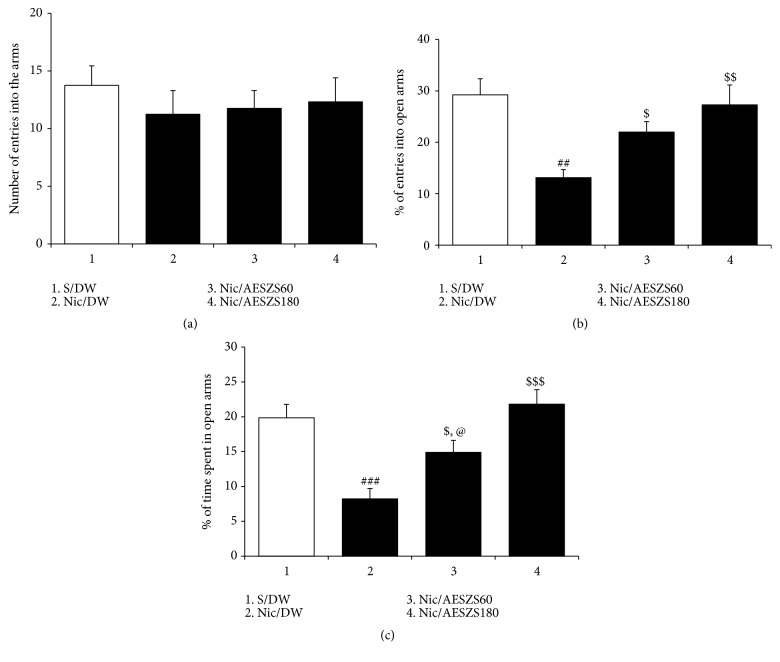
Effects of AESZS on NicW-induced anxiety-like behavior. Withdrawal from repeated Nic administrations induced obvious anxiety-like behavior in rats, but these behaviors were attenuated by AESZS treatment. All data are expressed as a mean ± SEM (*n* = 8). (a) The total number of entries into open and closed arms of the EPM by rats. (b) Percentage of numbers of entries into open arms of the EPM by rats. (c) Percentage of time spent in open arms by rats. S: saline; DW: distilled water; Nic: nicotine; NicW: nicotine withdrawal; AESZS: aqueous extract of Semen* Ziziphi Spinosae*; AESZS60: 60 mg/kg/d AESZS; AESZS180: 180 mg/kg/d AESZS. ^##^* p* < 0.01, ^###^* p* < 0.001 versus S/DW group; ^$^* p* < 0.05, ^$$^* p* < 0.01, ^$$$^* p* < 0.001 versus Nic/DW group; ^@^* p* < 0.05, versus Nic/AESZS180 group (one-way ANOVA followed by Newman-Keuls post hoc test).

In the present study, 4 d after the termination of Nic, rats showed anxiety-like behavior that was characterized by significant decreases in the numbers of entries into open arms and time spent in open arms compared to saline-treated controls [percentage of entries into open arms: F_(3,  28)_ = 6.64,* p *< 0.01; saline-treated control group (29.25 ± 3.11%,* n* = 8) versus Nic-treated control group (13.12 ± 1.57%,* n* = 8),* p *< 0.01; percentage of time spent in open arms: F_(3,  28)_ = 11.07,* p *< 0.001; saline-treated control group (19.84 ± 1.93%,* n* = 8) versus Nic-treated control group (8.22 ± 1.49%,* n* = 8),* p *< 0.001]. However, both the 60 mg/kg/d and 180 mg/kg/d AESZS doses significantly improved these anxiety indices in a dose-dependent manner [percentage of entries into open arms: Nic-treated control group versus Nic/AESZS60 group (21.98 ± 2.04%,* n* = 8),* p *< 0.05; Nic-treated control group versus Nic/AESZS180 group (27.29 ± 3.88%,* n* = 8),* p *< 0.01; percentage of time spent in open arms: Nic-treated control group versus Nic/AESZS60 group (14.89 ± 1.73%),* p *< 0.05; Nic-treated control group versus Nic/AESZS180 group (21.81 ± 2.08%),* p *< 0.001; Nic/AESZS60 group versus Nic/AESZS180 group,* p *< 0.05] (Figure 3).

### 3.2. Effects of AESZS on Plasma CORT Levels during NicW

**Figure 4:**
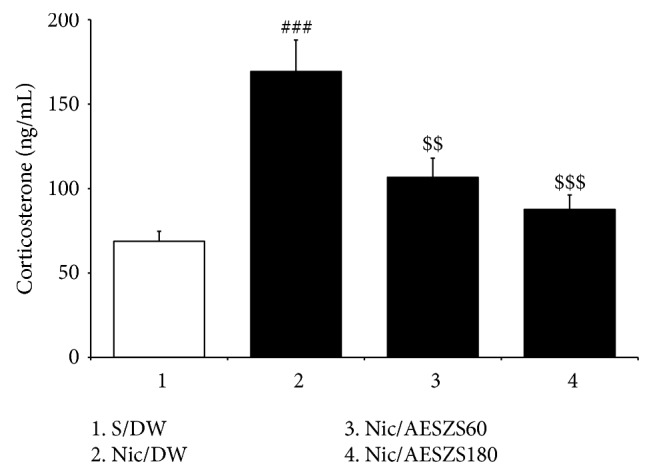
Effects of AESZS on plasma CORT levels during NicW. Withdrawal from repeated Nic administrations increased plasma CORT concentrations in rats, but these increases were prevented by AESZS treatment. All data are expressed as a mean ± SEM (*n* = 8). S: saline; DW: distilled water; Nic: nicotine; NicW: nicotine withdrawal; CORT: corticosterone; AESZS: aqueous extract of Semen* Ziziphi Spinosae*; AESZS60: 60 mg/kg/d AESZS; AESZS180: 180 mg/kg/d AESZS. ^###^* p* < 0.001 versus S/DW group; ^$$^* p* < 0.01, ^$$$^* p* < 0.001 versus Nic/DW group (one-way ANOVA followed by Newman-Keuls post hoc test).

Plasma CORT levels are an important hormonal indicator of anxiety in rats. Analyses of the ELISA results revealed that the plasma CORT levels of the Nic-withdrawn rats were significantly higher than those of saline-treated control rats [F_(3,  28)_ = 13.09,* p *< 0.001; saline-treated control group (68.71 ± 6.02, n = 8) versus Nic-treated control group (169.36 ± 18.65,* n* = 8),* p *< 0.001]. Consistent with the behavioral data, both doses of AESZS (60 mg/kg/d and 180 mg/kg/d) inhibited the increases in plasma CORT concentrations induced by NicW [Nic-treated control group versus Nic/AESZS60 group (106.69 ± 11.23,* n* = 8),* p *< 0.01; Nic-treated control group versus Nic/AESZS180 group (87.64 ± 8.55,* n* = 8),* p *< 0.001] (Figure 4).

### 3.3. Effects of AESZS on the Protein and mRNA Expressions of Amygdaloid CRF and CRF1R

**Figure 5:**
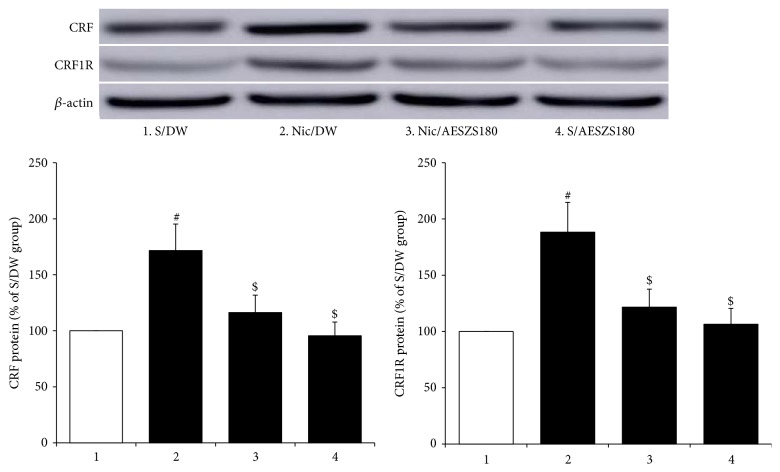
Effects of AESZS on CRF and CRF1R protein expressions in the CeA during NicW. Immediately after the EPM tests, the protein expressions of amygdaloid CRF and CRF1R were analyzed using Western blot assays. All data are expressed as a mean ± SEM (*n* = 4). S: saline; DW: distilled water; Nic: nicotine; AESZS: aqueous extract of Semen* Ziziphi Spinosae*; AESZS180: 180 mg/kg/d AESZS. ^#^* p* < 0.05, versus S/DW group; ^$^* p* < 0.05, versus Nic/DW group (one-way ANOVA followed by Newman-Keuls post hoc test).

**Figure 6:**
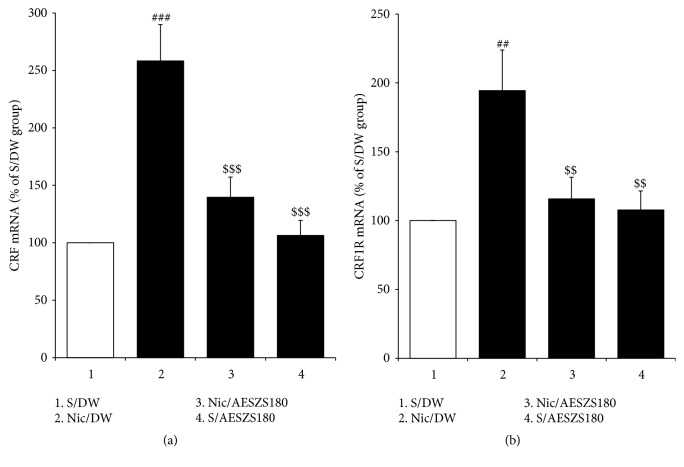
Effects of AESZS on the mRNA expressions of CRF and CRF1R in the CeA during NicW. Immediately after the EPM tests, the mRNA levels of amygdaloid CRF and CRF1R were examined using real-time PCR assays. All data are expressed as a mean ± SEM (*n* = 5). S: saline; DW: distilled water; Nic: nicotine; NicW: nicotine withdrawal; AESZS: aqueous extract of Semen* Ziziphi Spinosae*; AESZS180: 180 mg/kg/d AESZS. ^##^* p* < 0.01, ^###^* p* < 0.001, versus S/DW group; ^$$^* p* < 0.01, ^$$$^* p* < 0.001, versus Nic/DW group (one-way ANOVA followed by Newman-Keuls post hoc test).

To examine the possible involvement of the amygdaloid CRF-CRF1R system in the anxiolytic effects of AESZS during NicW, Western blot and real-time PCR analyses were conducted. Significant increases were observed in the protein expressions of both CRF and CRF1R in the CeA of Nic-treated controls compared to saline-treated controls [CRF: F_(3,  12)_ = 5.16,* p *< 0.05; saline-treated control group (100%,* n* = 4) versus Nic-treated control group (171.59 ± 23.67%,* n* = 4),* p *< 0.05; CRF1R: F_(3,  12)_ = 5.65,* p *< 0.05; saline-treated control group (100%,* n* = 4) versus Nic-treated control group (188.27 ± 26.53%,* n* = 4),* p *< 0.05]. However, these increases were effectively inhibited by treatment with 180 mg/kg/d AESZS [CRF: Nic-treated control group versus Nic/AESZS180 (116.39 ± 15.47%,* n* = 4),* p *< 0.05; CRF1R: Nic-treated control group versus Nic/AESZS180 (121.71 ± 15.94%,* n* = 4),* p *< 0.05] (Figure 5). Accordingly, real-time PCR analyses revealed that NicW significantly increased the mRNA expressions of both CRF and CRF1R in the CeA [CRF: F_(3,  16)_ = 14.55,* p *< 0.001; saline-treated control group (100%,* n* = 5) versus nicotine-treated control group (258.26 ± 31.74%,* n* = 5),* p *< 0.001; CRF1R: F_(3,  16)_ = 5.86,* p *< 0.01; saline-treated control group (100%,* n* = 5) versus nicotine-treated control group (194.41 ± 29.49%,* n* = 5),* p *< 0.01], but similar to the protein expressions, the administration of 180 mg/kg/d AESZS prevented the increases in mRNA expression [CRF: Nic-treated control group versus Nic/AESZS180 (139.61 ± 17.56%,* n* = 5),* p *< 0.001; CRF1R: Nic-treated control group versus Nic/AESZS180 (115.73 ± 15.72%,* n* = 5),* p *< 0.01] (Figure 6). No significant changes were observed in either the protein or mRNA expressions of CRF and CRF1R in the CeA when treated with 180 mg/kg/d of AESZS alone (Figures 5 and 6).

### 3.4. Effects of Post-AESZS Infusions of CRF into the CeA on the Anti-Anxiety Actions of AESZS

**Figure 7:**
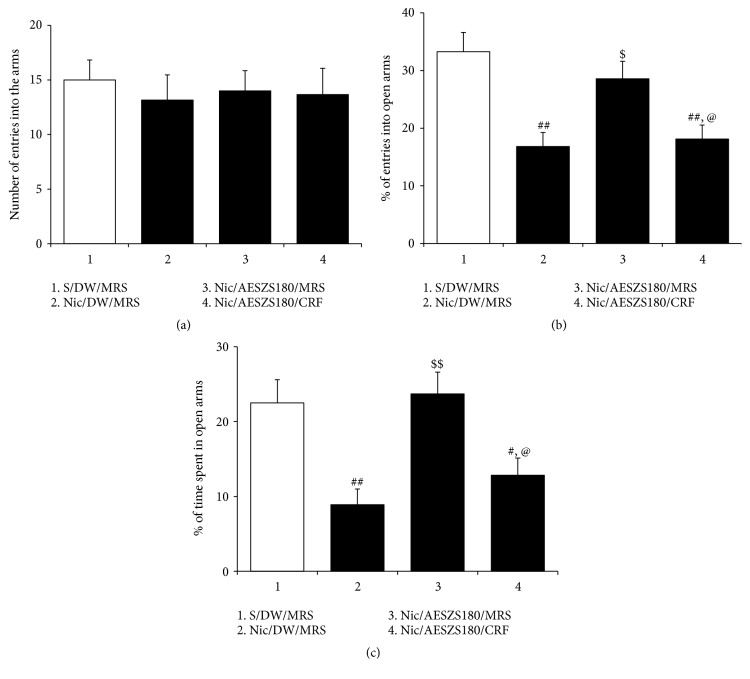
Effects of post-AESZS infusions of CRF into the CeA on the anti-anxiety actions of AESZS. At 60 min after the fourth 180 mg/kg/d AESZS, the rats received bilateral intra-CeA infusions of CRF and then were tested in the EPM to evaluate anxiety-like behavior. (a) The total number of entries into open and closed arms of the EPM by rats. (b) Percentage of numbers of entries into open arms of the EPM by rats. (c) Percentage of time spent in open arms by rats. All data are expressed as a mean ± SEM (*n* = 6). S: saline; DW: distilled water; Nic: nicotine; MRS: modified Ringers' solution; AESZS: aqueous extract of Semen* Ziziphi Spinosae*; AESZS180: 180 mg/kg/d AESZS. ^#^* p* < 0.05, ^##^* p* < 0.01, versus S/DW/MRS group; ^$^* p* < 0.05, ^$$^* p* < 0.01 versus Nic/DW/MRS group; ^@^* p* < 0.05 versus Nic/AESZS180/MRS group (one-way ANOVA followed by Newman-Keuls post hoc test).

To confirm whether the anxiolytic effects of AESZS during NicW was mediated via the CRF/CRF1R pathway in the CeA, an intra-CeA infusion of CRF was administered after the fourth AESZS treatment and the rats were tested in the EPM. Similar to the abovementioned EPM findings, Nic-withdrawn rats exhibited anxiety-like behavior that was ameliorated by treatment with 180 mg/kg/d AESZS [percentage of entries into open arms: F_(3,  20)_ = 7.96,* p *< 0.01; saline/DW/MRS group (33.25 ± 3.37 %,* n* = 6) versus Nic/DW/MRS group (16.83 ± 2.42%,* n* = 6),* p *< 0.01; Nic/DW/MRS group versus Nic/AESZS180/MRS group (28.58 ± 3.03%,* n* = 6),* p *< 0.05; percentage of time spent in open arms: F_(3,  20)_ = 7.58,* p *< 0.01; saline/DW/MRS group (22.50 ± 3.09%,* n* = 6) versus Nic/DW/MRS group (8.89 ± 2.10%,* n* = 6),* p *< 0.01; Nic/DW/MRS group versus Nic/AESZS180/MRS group (23.69 ± 2.92%,* n* = 6),* p *< 0.01]. In contrast, a post-AESZS infusion of CRF into the CeA abolished the anxiolytic effects of 180 mg/kg/d of AESZS [percentage of entries into open arms: Nic/AESZS180/MRS group versus Nic/AESZS180/CRF group (18.11 ± 2.42%,* n* = 6),* p *< 0.05; percentage of time spent in open arms: Nic/AESZS180/MRS versus Nic/AESZS180/CRF group (12.83 ± 2.31%,* n* = 6),* p *< 0.05](Figure 7).

### 3.5. Effects of AESZS on NE and MHPG Levels in the CeA during NicW

**Figure 8:**
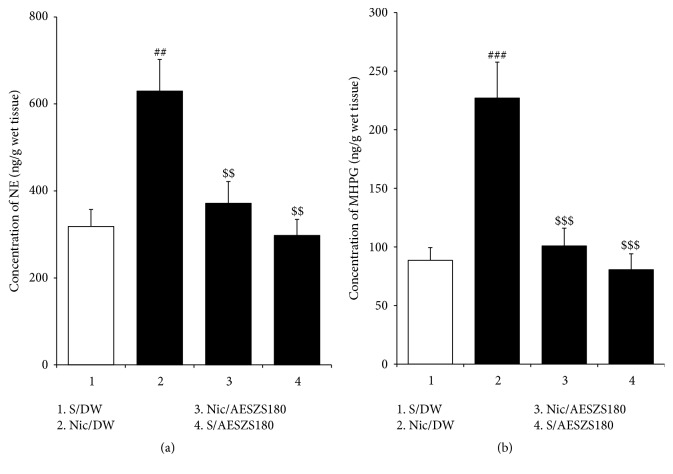
Effects of AESZS on NE and MHPG levels in the CeA during NicW. Immediately after EPM tests, the amygdaloid levels of NE and MHPG were determined using HPLC analysis. All data are expressed as a mean ± SEM (*n* = 6). S: saline; DW: distilled water; Nic: nicotine; AESZS: aqueous extract of Semen* Ziziphi Spinosae*; AESZS180: 180 mg/kg/d AESZS. ^##^* p* < 0.01, ^###^* p* < 0.001, versus S/DW group; ^$$^* p* < 0.01, ^$$$^* p* < 0.001, versus Nic/DW group (one-way ANOVA followed by Newman-Keuls post hoc test).

Alterations in NE and MHPG concentrations indicate a state of noradrenergic activation. In the present study, HPLC analyses revealed that NicW significantly increased both NE and MHPG levels [NE: F_(3,  20)_ = 8.80,* p *< 0.001; saline/DW group (318.23 ± 39.14,* n* = 6) versus Nic/DW group (629.67 ± 72.48,* n* = 6),* p *< 0.01; MHPG: F_(3,  20)_ = 12.90,* p *< 0.001; saline/DW group (88.62 ± 10.72,* n* = 6) versus Nic/DW group (226.95 ± 30.71,* n* = 6),* p *< 0.001], but these increases were inhibited by treatment with 180 mg/kg/d AESZS [NE: Nic/DW group versus Nic/AESZS180 group (371.46 ± 50.35,* n* = 6),* p *< 0.01; MHPG: Nic/DW group versus Nic/AESZS180 group (100.82 ± 15.20,* n* = 6),* p *< 0.001]. The administration of 180 mg/kg/d AESZS alone slightly decreased amygdaloid NE and MHPG levels but the difference was not statistically significant (Figure 8).

## 4. Discussion

The present study demonstrated that 60 mg/kg/d and 180 mg/kg/d AESZS once a day for 4 d during NicW dose-dependently alleviated NicW-induced anxiety-like behavior in rats and that 180 mg/kg/d AESZS reduced NicW-induced increases in plasma CORT concentrations. Additionally, Nic-withdrawn rats in the present study exhibited increases in the protein and mRNA expressions of both CRF and CRF1R in the CeA, while the administration of 180 mg/kg/d AESZS effectively prevented these increases. Moreover, 180 mg/kg/d AESZS inhibited NicW-induced increases in amygdaloid NE and MHPG levels. Taken together, these results suggest that AESZS attenuated NicW-induced anxiety in rats by improving CRF/CRF1R and noradrenergic signaling in the CeA.

Clinical and preclinical studies have reported that withdrawal from repeated exposure to Nic generates substantial levels of anxiety [11, 27]. This behavioral phenotype was evident in the EPM tests conducted in the present study, as the rats had decreased numbers of open-arm entries and spent less time in the open arms of the EPM compared to their Nic-spared counterparts. However, 60 mg/kg/d and 180 mg/kg/d AESZS reversed these decreases as well as the amount of time spent in open arms in a dose-dependent manner. These results indicate that AESZS can block NicW-induced anxiety if administered during NicW.

The crude extracts and bioactive components of SZS are well known to have anxiolytic effects. For example, the methanol extract of SZS increased the number of entries into open arms and time spent in open arms by mice in EPM tests [28]. Sanjoinine A, which is an alkaloid compound of SZS, exerted anxiolytic effects in mice in the EPM and open-field tests [29]. Furthermore, the oral administration of spinosin, which is a major flavonoid in SZS, produced anxiolytic effects in mice in EPM and light/dark box tests via the modulation of GABAa and 5-HT1a transmissions [23]. It is noteworthy that these studies investigated the innate fear and anxiety that animals have for open fields and elevated spaces, rather than anxious states precipitated by stress-induced pathophysiological changes in neuroendocrine systems. A preliminary experiment in the present study found that 180 mg/kg/d AESZS increased the number of entries into open arms and time spent in the open arms of the EPM in naive rats, indicating that this treatment had an inhibitory effect on innate anxiety. NicW is a stressor that induces dysregulation of the central nervous system in rats and leads to a pathophysiological state of anxiety [30]. Therefore, the present results that AESZS alleviated NicW-induced anxiety suggest that SZS was effective for both innate anxiety and noxious stimulus-induced anxiety behaviors as well.

Anxiety-like behavior is the manifestation of disordered stress hormone response in the body that is usually characterized by elevations in plasma CORT secretion [31]. In the present study, ELISA analyses revealed that Nic-withdrawn rats had higher plasma CORT concentrations than saline-treated control rats and that this increase was reversed by treatment with 180 mg/kg/d AESZS. These results suggest that AESZS can rectify hormonal disorders due to NicW, endocrinologically support the inhibitory effects of AESZS on anxiety-like behavior and further implicate the CRF-adrenocorticotropic hormone (ACTH)-CORT axis in this process.

CRF is a major stress hormone that initiates activation of the CRF-ACTH-CORT axis and orchestrates the neuronal and hormonal responses to stress. CRF is synthesized and released by CRF-containing neurons in the limbic system, including the CeA, where dysregulation of this system is thought to be the most important cause underlying anxiety induced by withdrawal from drugs of abuse [32]. CRF acts by binding to its two receptors, which are designated as CRF1R and CRF2R. CRF1R has a high affinity for CRF and is widely distributed throughout the brain, particularly in limbic regions such as the hippocampus, hypothalamus, and amygdala where CRF/CRF1R signaling mediates emotional stress responses, including anxiety [33]. In contrast, CRF2R has a low affinity for CRF and is discretely located in specific brain regions, such as the dorsal raphe nucleus and periaqueductal gray, and its activation typically counteracts the stress responses triggered by CRF/CRF1R transmission [34].

Abstinence from drugs of abuse leads to overexcitation within the amygdaloid CRF/CRF1R system [35], and previous evidence suggests that this system is primarily responsible for withdrawal-induced anxiety. Amygdaloid CRF1R, but not CRF2R, mediates anxiety-like behavior in mouse EPM tests [36, 37] and activation of CRF1R, but not CRF2R, in the CeA affects ethanol withdrawal-induced anxiety in rats [38]. A previous study by our research group revealed that ethanol withdrawal increases CRF mRNA expressions in the rat CeA [39]. In the present study, real-time PCR and Western blot analyses displayed increased protein and mRNA expressions for both CRF and CRF1R in the CeA of rats undergoing NicW, which are consistent with the studies done by others showing that NicW robustly increases the mRNA levels of CRF and CRF1R in the CeA [40]. Meanwhile, the same PCR and Western blot analyses also revealed that treatment with 180 mg/kg/d AESZS during NicW attenuated all of these increases. These results suggest that AESZS can suppress NicW-stimulated excessive excitement of the CRF/CRF1R system in the CeA and indicate the possible linkage of the anxiolytic effect of AESZS during NicW with its action on amygdaloid CRF/CRF1R signaling.

To further pharmacologically determine this linkage, in the present study, post-AESZS infusions of CRF (a preferential CRF1R agonist) were introduced into the CeA, and it was found that the CRF almost completely abolished the anxiolytic effects of 180 mg/kg/d AESZS during NicW. These results collectively suggest that the suppressive effect of AESZS on the CRF/CRF1R system in the CeA reverses the oversecretion of plasma CORT and inhibits anxiety-like behavior during NicW. It is worth noting that mechanistic studies of SZS pharmacology have so far focused on investigating the involvement of classic neurotransmitter systems such as GABA, glutamate, and 5-HT [19, 20]. Therefore, the present study is the first experimental observation to report that SZS acts on the central CRF/CRFR system (neuropetidergic system) as a pharmacological target.

Excessive activation of the noradrenergic system in the CeA is also critical for anxiety induced by withdrawal from drugs of abuse [7], which is, at least in part, accomplished via the CRF system. Ultrastructural and pharmacological analyses have revealed that CRF neurons in the CeA are targeted by noradrenergic afferents [41], that NE is a secretagogue of CRF, and that the activation of *β*-adrenergic receptors appears to stimulate CRF gene expression in the extended amygdala [15, 42]. Our previous studies have shown ethanol withdrawal simultaneously elevates noradrenergic transmission and CRF mRNA expression in the CeA [17, 39]; in addition, the studies done by others have also identified the parallel functional promotion of the noradrenergic and CRFergic systems in the CeA following the cessation of the chronic abuse of drugs [43, 44]. Accordingly, in the present study, NicW significantly increased both NE and MHPG levels in the CeA, whereas the administration of 180 mg/kg/d AESZS prevented these increases. Taken together, these results suggest that AESZS treatment during NicW curbs NicW-induced amygdaloid noradrenergic overactivation, which in turn blocks excessive excitement within the CRF/CRF1R pathway and ultimately eliminates anxiety-like behavior.

## 5. Conclusions

The present study demonstrated that AESZS treatment at 60 and 180 mg/kg/d dose-dependently attenuated NicW-induced anxiety and that 180 mg/kg/d AESZS suppressed the oversecretion of plasma CORT during NicW. Additionally, AESZS blocked NicW-induced increases in the protein and mRNA expressions of both CRF and CRF1R and reduced increases in the levels of NE and MHPG in the CeA. These results suggest that SZS can prevent NicW-induced anxiety via improvements in CRF/CRF1R and noradrenergic transmissions in the CeA.

Meanwhile, these results also indicate that SZS is a promising herbal candidate for treating NicW-induced anxiety and further aiding smoking cessation, but, in the present study, the dose-response relationship of AESZS, its ED50 and LD50 were not examined. Therefore, a future project must be done to identify which components of AESZS are essential to its anxiolytic effects during NicW and takes these components as a standard to determine the pharmacodynamics and pharmacokinetics of AESZS.

## Data Availability

The data supporting the conclusions of the present study are properly analyzed and included in Results section and are available from the corresponding author upon reasonable request.
